# Implementation of back at work after surgery (BAAS): A feasibility study of an integrated pathway for improved return to work after knee arthroplasty

**DOI:** 10.1002/msc.1633

**Published:** 2022-05-04

**Authors:** Daniël O. Strijbos, Geert van der Sluis, Tim A. E. J. Boymans, Stephan de Groot, Simon Klomp, Carolien M. Kooijman, Michiel F. Reneman, P. Paul F. M. Kuijer

**Affiliations:** ^1^ Department of Public and Occupational Health Amsterdam UMC University of Amsterdam Amsterdam the Netherlands; ^2^ Amsterdam Public Health Research Institute Amsterdam the Netherlands; ^3^ Research Programs Musculoskeletal Health Sports and Rehabilitation & Development Amsterdam Movement Sciences the Netherlands; ^4^ Department of Health Innovations Nij Smellinghe Hospital Drachten Drachten the Netherlands; ^5^ Hanze University of Applied Sciences Groningen Groningen the Netherlands; ^6^ Maastricht UMC + Maastricht the Netherlands; ^7^ Elabo Apeldoorn the Netherlands; ^8^ a.s.r. Insurances Utrecht the Netherlands; ^9^ Department of Orthopedics Nij Smellinghe hospital Drachten 9202 NN Drachten Compagnonsplein 1 the Netherlands; ^10^ Department of Rehabilitation University Medical Center Groningen University of Groningen Groningen the Netherlands

**Keywords:** feasibility studies, health plan implementation, knee arthroplasty, occupational health service, orthopedics, physical therapy modalities, return to work

## Abstract

**Purpose:**

Optimizing return to work after knee arthroplasty is becoming more important because of the growing incidence of KA among workers and poor return to work outcomes. The purpose of this study is to investigate the feasibility of Back At work After Surgery (BAAS): an integrated clinical pathway for return to work after knee arthroplasty.

**Method:**

Working patients who received unicompartmental knee arthroplasty (UKA) or total knee arthroplasty (TKA) between January 2021 and November 2021, younger than 65 years and motivated to return to work were eligible to participate. Feasibility was investigated on five domains: reach, dose delivered, dose received, fidelity and patients’ attitudes. These outcomes were obtained by a patient‐reported questionnaire and an interview with the occupational case manager and medical case manager.

**Results:**

Of the eligible 29 patients, eleven were willing to participate (response rate 38%; due to travel distance to and from the hospital). The dose delivered was between 91 and 100%, except information given about return to work from the orthopedic surgeon which was 18%. The dose received was 100%. For fidelity, case managers reported nine shortcomings for which five solutions were mentioned. In terms of patients’ attitude, all patients were satisfied and one patient mentioned an improvement.

**Conclusions:**

In terms of reach, participation was low: only 29%. The BAAS clinical pathway seems feasible based on dose delivered, dose received, fidelity and patient attitudes. The next step is to assess the effectiveness of the BAAS clinical pathway for return to work.

## 1 INTRODUCTION

For patients, return to work (RTW) after knee arthroplasty (KA) is becoming more important due to the growing incidence of KA, especially among patients of working age (Ackerman et al., 2019; Culliford et al., 2015; Kurtz et al., 2007; Otten et al., 2010; Price et al., 2018). Although pain relief and knee function are satisfactory after KA, RTW among patients is relatively low, about 50% within 6 months after KA (Hylkema et al., 2021), and 68% do not return to work after 2 years (Kievit, van Geenen, et al., 2014).

A closer collaboration between professionals in surgical care (e.g., orthopaedic surgeons, physical therapists) and occupational care (e.g., occupational physicians) might improve RTW (Hylkema et al., 2022). Unfortunately, proven effective interventions are not available yet for these patients (Coenen et al., 2020; Kuijer et al., 2018).

We developed Back At work After Surgery (BAAS): an integrated clinical pathway for improved RTW after KA. This newly designed pathway follows the recommendations of the clinical guideline to optimise work participation by timely combination of medical and occupational care (Daley et al., 2021).

To the best of our knowledge, this is the first initiative to evaluate the feasibility of work‐directed care among patients receiving KA.

## 2 METHOD

### 2.1 Study design and setting

A single centre study was performed with permission of the medical ethical committee of Nij Smellinghe hospital (reference ID: 17050/JvE/AB) and described using the STROBE statement (Vandenbroucke et al., 2007). KA was performed by three of the five orthopaedic surgeons in this regional hospital.

### 2.2 Participants

The following are the patient eligibility criteria: (1) being scheduled for UKA or TKA due to knee osteoarthritis between January and November 2021; (2) having paid work; and (3) willing to fully RTW. Exclusion criteria were (1) receiving another surgical intervention within 1 year; (2) having a major mental disorder; (3) insufficiently fluency in Dutch; or (4) not willing to receive physical therapy in Nij Smellinghe hospital. Eligible patients were informed by telephone about the study and received an information letter, informed consent and an infographic of the BAAS clinical pathway at home (Appendix I). Patients were called 1 week later to answer any additional questions about the study and were asked if they wanted to participate by signing the informed consent.

### 2.3 Intervention: The BAAS clinical pathway

**FIGURE 1:**
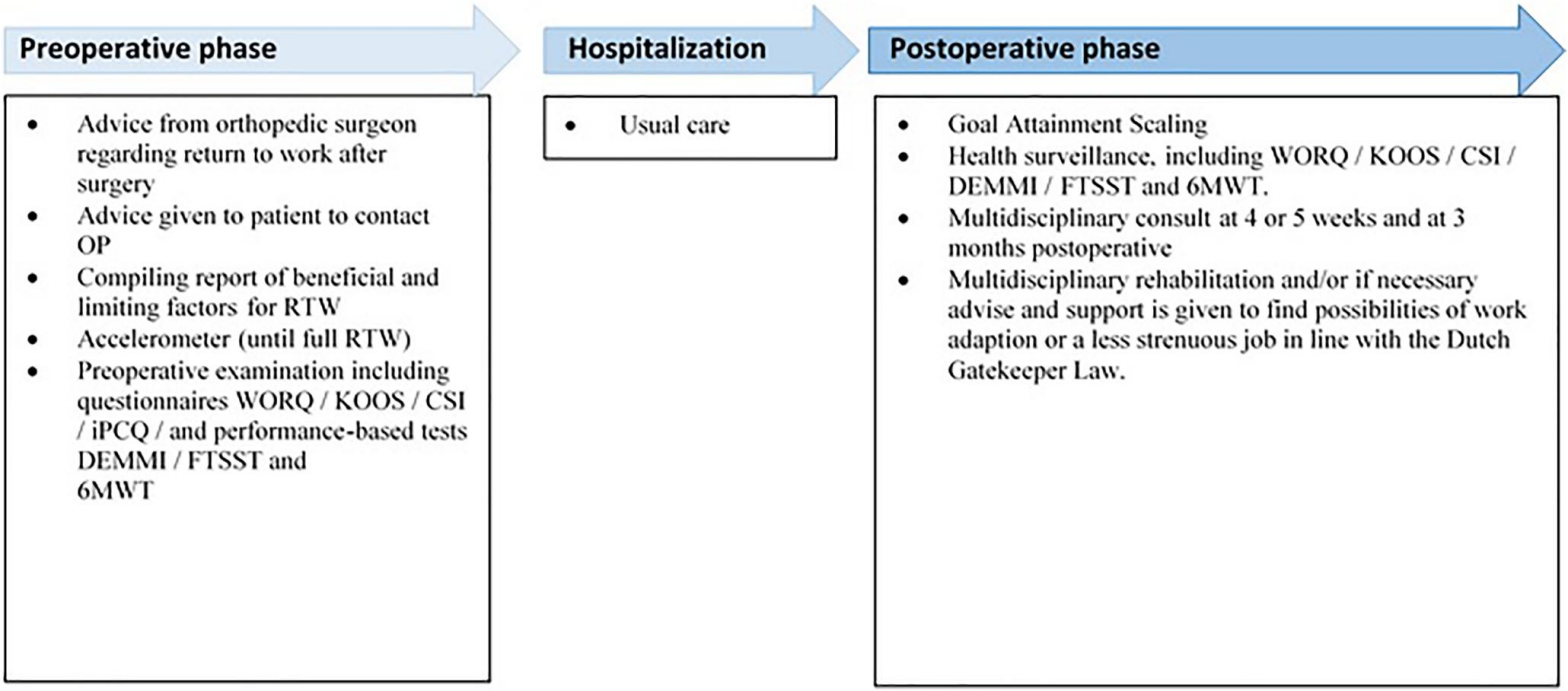
BACK@WORK clinical pathway. Abbreviations: 6MWT, 6‐min walk test; CRT, Chair Rise Time; CSI, Central Sensitisation Inventory; DEMMI, de Morton Mobility Index; FTSST, Five Time Sit to Stand; iPCQ, iMTA Productivity Cost Questionnaire; KOOS, Knee Osteoarthritis Outcome Score; OP, occupational physician; RTW, return to work; WORQ, Work, Osteoarthritis and Joint‐Replacement Questionnaire

The orthopaedic surgeon preoperatively provided information about time to RTW and prognostic factors for delayed RTW (Figure 1) (Kuijer et al., 2016; van Leemput et al., 2021) and recommended that the patient consult the occupational physician before surgery. Next, the patient was referred to the occupational case manager (OCM, occupational assessor) to compile a report of beneficial and limiting factors regarding RTW after KA.

Next, the patient was referred to a MCM for a preoperative examination to address the patient's expectations and set a benchmark for physical functioning (Krysa et al., 2022). The patient also filled in questionnaires and performed three tests to evaluate functioning (Table 1 and Figure 1) (Bouwmans, 2015; Csuka et al., 1985; Enright et al., 2003; Jans et al., 2011; Kievit, 2014; Kregel, 2016; Roos, 1998). Moreover, the patient was given information about the perioperative care and received an accelerometer (PAM 2.0) and access to the Atris platform to assess the movement data (Peercode B.V.Slootmakers, 2009). A week later, the MCM called the patient to discuss the physical activity assessed by the accelerometer and gave advice regarding the preferred physical preparation, like practicing to walk with an aid and trying to adhere to the World Health Organization's activity guideline (Larsen et al., 2022; WHO, 2020). The findings of the preoperative examination were used as baseline measurements for goalsetting in the postoperative rehabilitation and, not as an outcome for this feasibility study. During the hospitalisation, the patients received care according to the KA fast‐track principles (Altman et al., 2019).

**TABLE 1:** Questionnaires and functional tests used to measure recovery

	Measures	Total score	Lower score indicates
Questionnaires			
Work, osteoarthritis and joint‐replacement questionnaire (WORQ)	Thirteen 0–4 scale questions	0‐100 scale total score	More disability in work‐related and knee demanding activities
Knee injury and osteoarthritis outcome score (KOOS)	Thirteen 0–4 scale questions regarding work.	0‐100 scale total score in five domains (KA related symptoms, pain, activities, sport participation and quality of life).	Less experienced disabilities in given domains.
Central sensitisation inventory (CSI)	Twenty‐five 0–4 scale questions	0‐100 scale total score	Less chance of developing central sensitisation related disabilities.
iMTA productivity cost questionnaire (iPCQ)	Eleven questions regarding health and work	‐	‐
Functioning testing			
de Morton mobility index (DEMMI)	Eleven 0–1 and four 0–2 scale questions	0‐100 scale total score	Less able to perform mobility skills
Five Time sit to stand Test (FTSST)	Time in seconds	Time in seconds	Less able to perform mobility skills
6‐min walk test (6MWT)	Time in seconds	Time in seconds	Less able to perform mobility skills

Postoperatively, the patient received physical therapy according to the Royal Dutch Society for Physiotherapy guideline, starting sessions twice a week (van Doormaal et al., 2020). The first sessions were at the patient's home to train activities of daily living, such as climbing stairs. These sessions started 24–48 h after discharge from the hospital until about week 4. Next, physical therapy was once or twice a week, depending on the patient's needs, at the outpatient facility within Nij Smellinghe hospital. Goals shifted from activities of daily living to activities necessary for RTW. Goals were formally monitored every 6 weeks by Goal Attainment Scaling (GAS, Figure 1). Progress was evaluated by questionnaires (WORQ, KOOS, and CSI) and functional tests (DEMMI, FTSST and 6MWT) every 6 weeks and accelerometry on a weekly basis (Figure 1). Stop criteria were full RTW with a maximum follow‐up of 2 years. A multidisciplinary consult (MDC) was held the fourth or fifth week after KA. The patient, the physical therapist, MCM, OCM and occupational physician were invited and the progress in recovery was discussed including the RTW plan according to the Dutch Gatekeeper Improvement Act (Figure 1). If the patient had a job which he likely could not return to (e.g., because of high knee demands), the possibility of work adaptions or even the topic of finding a less strenuous job were discussed (Figure 1). The MDC was continued if indicated. If, after 3 months, a delayed recovery was seen based on patient's experience, the measured data and the expert opinions of MCM, OCM and occupational physician, the patient was referred to a multidisciplinary rehabilitation assessment (Figure 1). Here, the patient was examined by a rehabilitation physician, occupational specialist, physical therapist and psychologist to assess barriers for delayed RTW and the patient's eligibility for an interdisciplinary vocational rehabilitation programme. If eligible, the patient then received this programme (Beemster et al., 2021).

### 2.4 Feasibility outcomes

To assess feasibility, five outcomes were analysed: reach, dose received, dose delivered, fidelity and patient attitudes (Linnan et al., 2002). To assess reach, the degree of participation by the patients, occupational physicians and employers was analysed. To assess dose delivered, the proportion of the intervention that was delivered to the participants by orthopaedic surgeons, OCM and MCM was analysed. For dose received, exposure and usage of the intervention components was measured using a self‐reported questionnaire filled in by the patients after they fully returned to work or 6 months after surgery (Appendix II) (Bowen et al., 2009). To assess fidelity, an interview was held with the MCM and OCM by a third author (PK). For each patient, it was discussed whether the intended care was given as planned. Lastly, patient attitudes were measured using a self‐reported questionnaire about the received care (Appendix II).

### 2.5 Sample size

We aimed to include 20 consecutive patients. This number was based on expectations that these 20 patients secure enough variability to determine whether the BAAS clinical pathway is feasible for all eligible patients with KA. To assess variability, prognostic factors for RTW of each patient were described, including preoperative sick‐leave duration, body mass index and physical demands of the job (Kuijer et al., 2016).

### 2.6 Statistical methods

Patient characteristics were described with median and interquartile range (IQR). Primary outcomes (reach, dose delivered, dose received, fidelity and patient attitudes) were described. Data were analysed using SPSS version 26.1.

## 3 RESULTS

### 3.1 Participants

**FIGURE 2:**
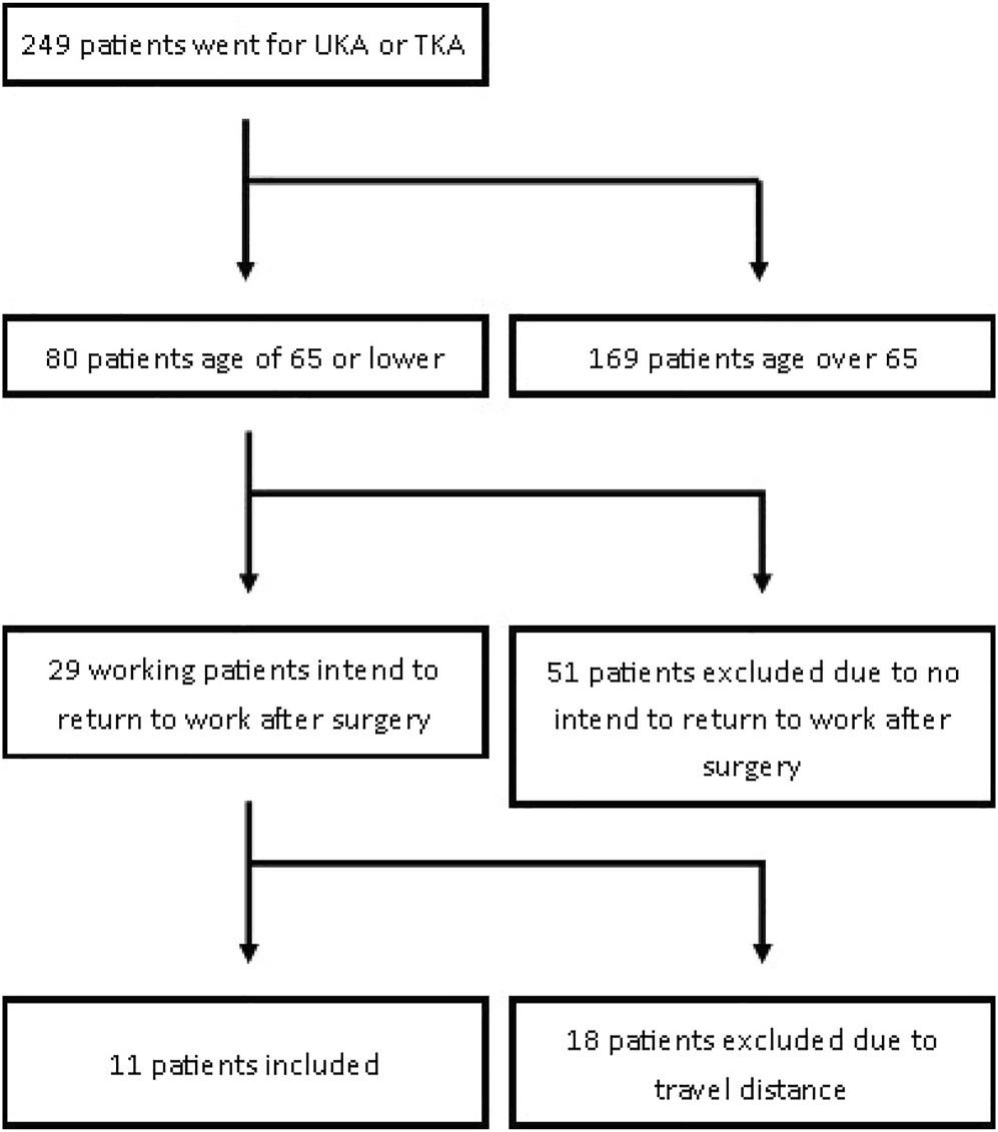
Flowchart of in‐ and exclusion. TKA, total knee arthroplasty; UKA, unicompartmental knee arthroplasty

In the study period, 249 patients received a TKA or UKA and 80 were younger than 65 years. Fifty‐one patients did not meet the inclusion or exclusion criteria, and 29 patients were asked to participate. 11 agreed to participate. The remaining 18 patients were not willing to participate due to long travel distance to the hospital (Figure 2).

### 3.2 Descriptive data

Of the 11 participants, the median age was 59 years, seven were female, and eight received TKA (Table 2). All patients fully returned to work within the first year, on average after 14 weeks after KA. One patient switched to another employer and started in a less demanding job. One patient was seen for a multidisciplinary rehabilitation assessment.

**TABLE 2:** Preoperative patient characteristics for participants in the Back At work After Surgery (BAAS) clinical pathway

	Median [Q1‐Q3]^a^ or number^b^ (percentage)	Missing data (percentage)
Age^a^	59 [57–60]	0
Sex^b^		0
‐ Male	4 (36%)	
‐ Female	7 (64%)	
Surgery^b^		0
‐ UKA	3 (27%)	
‐ TKA	8 (73%)	
Length of stay (days)^a^	2 [2–2.5]	0
Number of patients reported preoperative sick leave > 2 weeks*	1 (9%)	0
Knee demanding work^b^		0
‐ Low	2 (18%)	
‐ Medium	4 (36%)	
‐ High	5 (45%)	
Level of education^b^		0
‐ Low	5 (45%)	
‐ Medium	3 (27%)	
‐ High	3 (27%)	
Functioning^a^		
‐ DEMMI (range 0–100)	85 [85–100]	2 (18%)
‐ 6 MWT	407 [397–449]	2 (18%)
‐ 5 STST	11.4 [10.3–12.7]	2 (18%)
Patient reported outcomes^a^		
‐ CSI (range 0–100)	23 [16.5–31.5]	1 (9%)
‐ KOOS		1 (9%)
o Symptoms (range 0–100)	42.9 [33.9–51.8]	
o Pain (range 0–100)	47.2 [38.9–50]	
o Activities (range 0–100)	57.4 [52.2–64.0]	
o Sport participation (range 0–100)	5 [0–17.5]	
o Quality of life (range 0–100)	18.8 [6.3–28.1]	
‐ WORQ (range 0–52)	42 [36.5–58.7]	1 (9%)
Return to work^a^		
‐ Weeks to start return to work	6.4 [6–8.1]	0
‐ Weeks to fully return to work	12.4 [9.8–14.4]	0

Abbreviations: 5 STST, 5 time sit‐to‐stand test; 6MWD, 6 min walking test; CSI, Central Sensitisation Inventory; DEMMI, de Morton Mobility Index; KOOS, Knee Osteoarthritis Outcome Score; WORQ, Work, Osteoarthritis and joint‐Replacement Questionnaire; TKA, Total Knee Arthroplasty; UKA: Unicompartmental Knee Arthroplasty.

^a^ Numeric variables.

^b^ categorical variables.

### 3.3 Feasibility

All patients completed the feasibility questionnaire (Table 3). In terms of reach, 38% of the invited patients decided to participate. Regarding dose delivered, the orthopaedic surgeons informed patients only two times (18%) about RTW. All patients received the intended care. One patient did not have contact with an occupational physician and did not have contact with the employer about RTW. This was due to the fact that this patient was self‐employed.

**TABLE 3:** Feasibility outcomes of the Back At Work after Surgery (BAAS) clinical pathway

	Number (%)
Reach	
Number of patients who met inclusion criteria	29 (100)
Number of patients included	11 (38)
Number of cases where occupational physician was willing to participate	11 (100)
Number of cases where employer was willing to participate	11 (100)
Number of cases where OCM was involved	11 (100)
Number of cases where MCM was involved	11 (100)
Dose delivered (OS/MCM/OCM/OP/MDR)	
Information return‐to‐work from orthopaedic surgeon	2 (18)
Advice given to contact occupational physician before surgery	10 (100)
Invitation sent for preoperative screening by MCM	10 (91)
Set work‐related goal before surgery	10 (91)
Physical therapy received until full return to work	11(100)
Contact OCM	11 (100)
Reach out before surgery	4 (36)
Reach out after surgery	7 (64)
Multi‐disciplinary consult organised	11 (100)
Accelerometer and information about usage was given	11 (100)
Patients seen for quick scan before IVR	1 (100)
Dose received (patients)	
Information return‐to‐work from orthopaedic surgeon	2 (100)
Contact with occupational physician	10 (100)
Start before surgery	3 (30)
Start after surgery	10 (100)
Preoperative screening by MCM	10 (100)
Set work‐related goal before surgery	10 (100)
Physical therapy received until full return to work	11 (100)
Contact OCM	11 (100)
Before surgery	2 (50)
After surgery	10 (91)
Multi‐disciplinary consult	11 (100)
Contact employer about return‐to‐work	10 (100)*
Before surgery	3 (27)
After surgery	10 (100)*
Accelerometer	11 (100)
Worn daily	11 (100)
Fidelity	
Number of difficulties mentioned by OCM and MCM	9
Number of improvements mentioned by OCM and MCM	5
Patients' attitude	
Patient satisfied	11 (100)
Improvements mentioned	1 (9)

Aberrations: MCM, medical case manager; OCM, occupational case manager; IVR, nterdisciplinary vocational rehabilitation; OS, orthopaedic surgeon; OP, occupational physician.

In terms of fidelity, seven shortcomings in care were reported. For these shortcomings in care, OCM and MCM mentioned five possible improvements (Table 4).

**TABLE 4:** Shortcomings and solutions mentioned by medical and occupational case managers

Shortcomings	Solutions
• In multiple cases, it was hard to get in contact with the occupational physician (4 out of 11) or employer (3 out of 11);	• Compiling an information letter regarding the BAAS clinical pathway, which is sent to the OP and employer to improve communication with OP and employers;
• In three cases, the communication between OCM and MCM was not optimal given that the MCM was not informed when the report was finished or the OCM was not kept up to speed on cases regarding the RTW process;	• In case an OP is not involved, the OCM and MCM will advise the patient on RTW;
• The report made by OCM often came after surgery instead of before surgery (9 out of 11). This was due to the variability between the long waiting time before surgery during the SARS‐COVID‐19 outbreaks in the Netherlands and sudden short waiting times when surgery was possible to perform after the outbreak periods;	• investigate whether employers can join the MDO if patients provide informed consent regarding discussing their work‐related disabilities;
• The OCM had a hard time figuring out which patient had fully returned to work and therefore which cases could be closed;	• Organise a meeting every 2 weeks between OCM and MCM to discuss progress to improve communication between OCM and MCM;
• In one case, an occupational physician was not involved because the patient was self‐employed and had no contract with an occupational physician or health service. Therefore, no occupational physician was present during the RTW;	• Announce new patients to OCM directly after signing informed consent and implement a maximum period of 20 days between announcement and finishing the OCM report.
• Two patients went on holiday in the RTW period and therefore the rehabilitation and RTW process was postponed. This may have led to a longer RTW period;	
• The report of the OCM was not always up to date given that one patient was fired during the RTW period and one patient switched jobs before surgery while the report from OCM was already compiled, which delayed or complicated RTW;	

Aberrations: MCM, medical case manager; OCM, occupational case manager; OP, occupational physician; RTW, return to work.

Finally, all patients mentioned being satisfied with the BAAS clinical pathway. One patient was dissatisfied that the orthopaedic surgeon had not mentioned the high risk of no RTW due to his high knee‐demanding work.

## 4 DISCUSSION

### 4.1 Key results

The BAAS clinical pathway appeared to be feasible in terms of dose delivered, dose received, fidelity and patients' attitudes. The reach appeared low in comparison to other feasibility studies among patients receiving orthopaedic care reporting data about 63%, 59% and 30% (Kumar et al., 2020; Resnick et al., 2016; Vrenceanu et al., 2019). Our reach was 38%. This low reach was mainly due to the long travel distance to the hospital for physical therapy.

### 4.2 Interpretation

To increase reach, we suggest giving the physical therapy care from primary care physical therapists nearby the patient's home. This is also in line with the right care in the right place (https://english.zorginstituutnederland.nl/). To increase the dose delivered, we advise training the orthopaedic surgeons to inform patients better about RTW and relevant prognostic factors, in line with recent studies (Kuijer et al., 2016; Pahlplatz et al., 2021; van Leemput et al., 2021). Future studies should investigate whether these measures indeed improved the feasibility.

### 4.3 Generalisability

The BAAS clinical pathway seems feasible within the Dutch social security and care systems. Further research is needed to assess the effectiveness on RTW. This study will be performed with a second hospital in the Netherlands to overcome any bias introduced by the clinical involvement of the primary researcher. In comparison to other studies among working‐age patients with KA, the present patient characteristics were similar in terms of age, sex and educational level (Hylkema et al., 2021; van Leemput et al., 2021). An important prognostic factor for RTW for patients with KA are the physical job demands (van Leemput et al., 2021). Despite the low number of participants, the job demands ranged from low knee‐demanding desk work to high knee‐demanding work as a mechanic in which RTW appeared not possible. Lastly, the primary investigator fulfiled the roll of MCM. Thus, the expertise regarding RTW after KA might be higher than average. Therefore, future MCMs in other hospitals and clinics should be trained before implementing the BAAS clinical pathway. For the OCM, external validity is covered by using a service available nationwide due to the Dutch Gatekeeper Improvement Act.

### 4.4 Strengths and limitations

The BAAS clinical pathway is, to the best of our knowledge, the first clinical pathway to focus on RTW as the main outcome after KA (Coenen et al., 2020; Kuijer et al., 2018). Unfortunately, no data were available on the feasibility or effectiveness of the Occupational advice for Patients undergoing Arthroplasty of the Lower limb (OPAL studyBaker et al., 2020). Important similarities between OPAL and our pathway are an interdisciplinary approach and patient‐centred care. This is in line with the clinical guidance of Daley and colleagues on work participation (Daley et al., 2021). They advised a timely combination of medical rehabilitation and occupational RTW care, including the use of self‐reported and performance‐based measures. In the Netherlands, another intervention was evaluated for working‐age patients with KA, aiming at improved RTW, namely the ACTIVE trial (https://www.trialregister.nl/trial/8525) (Straat et al., 2020). This intervention combines referral to a case manager in the hospital or clinic and rehabilitation with personalised goals and eHealth. In contrast with the BAAS clinical pathway, the case manager in the ACTIVE trial does not actively enhance cooperation or communication between patient, employer, OCM and the other healthcare professionals.

This study had two important limitations. First, the number of participating patients did not reach 20, but only 11. This was mainly due to restrictions in number of KA in the hospital, related to the SARS‐COVID‐19 pandemic. Secondly, the willingness of primary physical therapy settings to participate in the present BAAS pathway instead of physical therapy given from the hospital is not investigated. Future studies should show whether this is feasible.

## CONFLICT OF INTEREST

All authors declare that they have no conflicts of interest.

## ETHICS STATEMENT

Our study was performed in accordance with the declaration of Helsinki and Directive 95/46/EG of the European Union regarding data protection. According to Dutch law, research with anonymised care data acquired by the physiotherapist treating the patient does not require approval from a medical ethical committee and is not subjected to a procedure for informed consent applied by the hospital. This standpoint was confirmed by the ethical committee of Nij Smellinghe hospital with respect to this particular study (reference ID: 17050/JvE/AB).

## AUTHOR CONTRIBUTIONS

All authors have made: (1) Substantial contributions to conception and design, acquisition of data, or analysis and interpretation of data; (2) Been involved in draughting the manuscript and revising it critically for important intellectual content; (3) Given final approval of the version to be published; and (4) Agreed to be accountable for all aspects of the work in ensuring that questions related to the accuracy or integrity of any part of the work are appropriately investigated and resolved.

## Supporting information

Figure S1

Table S1

## Data Availability

The data that support the findings of this study are available on request from the corresponding author. The data are not publicly available due to privacy or ethical restrictions.
