# Supplementary figures and images for: Implementation of back at work after surgery (BAAS): A feasibility study of an integrated pathway for improved return to work after knee arthroplasty
Source: Musculoskeletal Care. 2022 May 4;20(4):950–9. doi: 10.1002/msc.1633 (PMC10084307; doi:10.1002/msc.1633)

**Appendix I – Comic of the Back At work After Surgery (BAAS) clinical pathway**

**
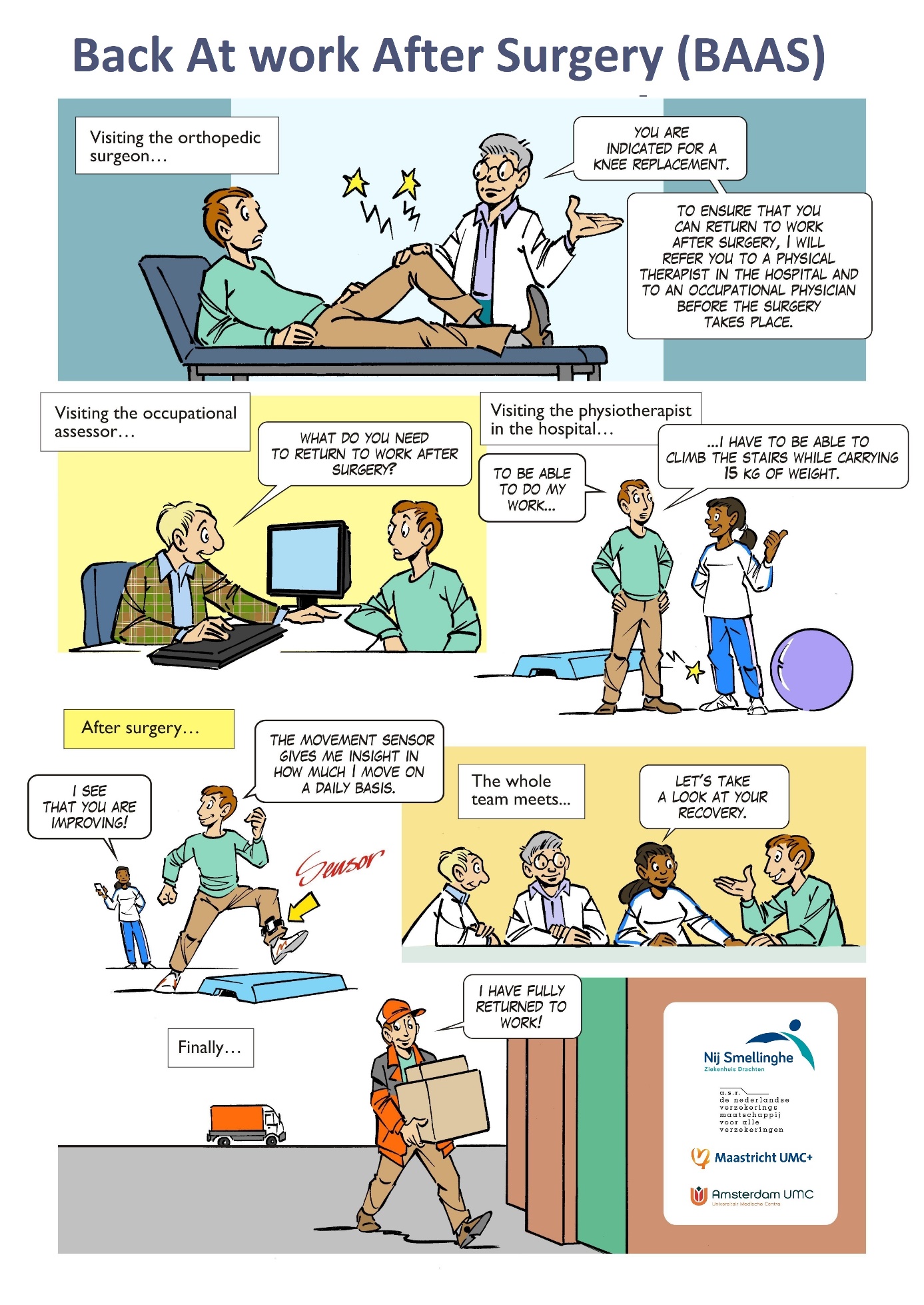
**

Supplement: Supplementary file 1 — Figure S1 [file MSC-20-950-s002.docx]
