# Supplementary material for: Implementation of back at work after surgery (BAAS): A feasibility study of an integrated pathway for improved return to work after knee arthroplasty
Source: Musculoskeletal Care. 2022 May 4;20(4):950–9. doi: 10.1002/msc.1633 (PMC10084307; doi:10.1002/msc.1633)
Supplement: Supplementary file 2 — Table S1 [file MSC-20-950-s001.docx]

**Appendix II – Self reported questionnaire**

|  |  | \|  \| \| --- \| \| Questionaire Back at work after surgery \| \|  \|   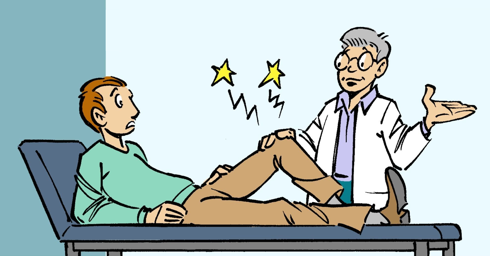 |
| --- | --- | --- | --- | --- | --- |

1. Before and after surgery you spoke to an orhopedic surgeon. What did he or she told you regarding return to work?

|  | The orthopedic surgeon did not mention anything about return to work |
| --- | --- |
|  | The orthopedic surgeon did mention the current study with the pupose of increasing the chance of early return to work after surgery. |
|  | Other, namely: Klik hier als u tekst wilt invoeren. |

1. Did you speak to an occupational physician before or after surgery?

|  | I had contact with an occupational physician before and after surgery |
| --- | --- |
|  | I had contact with an occupational physician before surgery |
|  | I had contact with an occupational physician after surgery |
|  | I did not have contact with an occupational physician 🡪 skip question 3 |

1. What topics did you discuss with your occupational physician?

|  | The occupational physician spoke with me about expectations regarding return to work after surgery. |
| --- | --- |
|  | The occupational physician spoke with me about the possibilities of adjustment which can be made at work to enhance return to work after surgery. |
|  | The occupational physician spoke with me about which factor regarding work need special attention to enhance return to work after surgery. |
|  | Other, namely: Klik hier als u tekst wilt invoeren. |

1. Did tou have a consult with a physicial therapist within the hospital?

|  | Yes, during this meeting we set a work related treatment goal. |
| --- | --- |
|  | Yes, during this meeting we did not set a work related treatment goal. |
|  | No |

1. Did you receive physical therapy untill you had fully returned to work?

|  | Yes |
| --- | --- |
|  | No |
|  | Other, namely: Klik hier als u tekst wilt invoeren. |

1. Did you receive the accelerometer?

|  | Yes |
| --- | --- |
|  | No |
|  | Other, namely: Klik hier als u tekst wilt invoeren. |

1. How often did you wear the accelrometer?

|  | Every day |
| --- | --- |
|  | At least twice a week |
|  | At least once a week |
|  | At least once a month |
|  | Other, namely: Klik hier als u tekst wilt invoeren. |

1. Did you have contact with an occupational assessor?

|  | Yes. I have had contact with an occupational assessor before and after surgery. |
| --- | --- |
|  | I Yes. I have had contact with an occupational assessor before surgery. |
|  | Yes. I have had contact with an occupational assessor after surgery. |
|  | No, I did not have any contact with an occupational assessor |

1. Did you have contact with your employer regarding surgery?

|  | Yes, before and after surgery |
| --- | --- |
|  | Yes, before surgery |
|  | Yes, after surgery |
|  | No |

1. Did a consultation took place five weeks after surgery with you, the occupational assessor, occupational phycisian and the physiotherapist?

|  | Yes |
| --- | --- |
|  | No |
|  | Other, namely: Klik hier als u tekst wilt invoeren. |

1. What did you experience as good about this trajectory, and what could be better?

|  | Klik hier als u tekst wilt invoeren. |
| --- | --- |
